# Comparison of free vascularized fibular grafts and the Masquelet technique for the treatment of segmental bone defects with open forearm fractures: a retrospective cohort study

**DOI:** 10.1186/s10195-024-00787-x

**Published:** 2024-09-28

**Authors:** Ming Zhou, Yunhong Ma, Xueyuan Jia, Yongwei Wu, Jun Liu, Yapeng Wang, Peng Wang, Junhao Luo, Fang Lin, Jianbing Wang, Yongjun Rui

**Affiliations:** Department of Orthopaedic Surgery, Wuxi Ninth People’s Hospital Affiliated to Soochow University, Liangxi Road, No. 999, Binhu District, Wuxi, Jiangsu China

**Keywords:** Free vascularized fibular grafts, Forearm, Bone defect, Masquelet technique

## Abstract

**Purpose:**

Severe open forearm fractures commonly involve segmental bone defects. Although several methods have been proposed to treat segmental bone defects with such fractures, research comparing the radiological and clinical outcomes of free vascularized fibular grafts (FVFG) and the Masquelet technique (MT) is rare.

**Methods:**

Data on 43 patients with open forearm fractures and segmental bone defects treated surgically in our hospital from January 2005 to January 2021 were retrospectively analyzed, and these patients were divided into an FVFG group (18 cases) and an MT group (25 cases). Clinical and radiological evaluations were performed regularly, and the minimum follow-up was 18 months.

**Results:**

All 43 patients were followed up for 18 to 190 months, with a mean of 46.93 months. The mean follow-up time was significantly longer in the FVFG group than in the MT group (*p* = 0.000). Bone healing time was 3–16 months, with a mean of 4.67 months. The QuickDASH score at the last follow-up was 0–38.6, with a mean of 17.71, and there was no statistically significant difference between the two groups. Operative time, hospital stay, and intraoperative bleeding for bone defect reconstruction were higher in the FVFG group compared to the MT group (*p* = 0.000), whereas the number of procedures was lower in the FVFG group than in the MT group (*p* = 0.035).

**Conclusions:**

FVFG and the MT showed satisfactory clinical results for segmental bone defects of the forearm. Compared with FVFG, the MT exhibited a lower operative time, hospital stay, and intraoperative bleeding.

**Level of evidence:**

Level IV.

*Trial registration* This study was registered in the Chinese Clinical Trial Registry (registration no. ChiCTR2300067675; registered 17 January 2023), https://www.chictr.org.cn/showproj.html?proj=189458.

**Supplementary Information:**

The online version contains supplementary material available at 10.1186/s10195-024-00787-x.

## Introduction

High-energy forearm fractures are typically severe and complicated, with extensive soft-tissue injuries and segmental bone defects. The surgical treatment of open forearm fractures is clinically demanding due to the intricate anatomy of the forearm, given that the length and angulation of both the ulnar and radial bones need to be restored [1, 2]. Repairing extended segmental bone defects remains clinically challenging [3], and the repair methods include conventional bone grafting, distraction osteogenesis, vascularized bone grafting (e.g., free vascularized fibular grafts), and the Masquelet technique (MT).

Vascularized bone graft is the classical surgical approach to covering the trauma surface. Bone segments, along with blood vessels, skin, subcutaneous tissue, and muscles, are harvested using microsurgical techniques. The fibula, iliac crest, and ribs are the most commonly dissected bone segments for bone grafts [4]. Among them, a fibular bone graft is the most commonly used option. The use of free vascularized fibular grafts (FVFG) in post-traumatic upper-extremity reconstruction surgery was proposed by Taylor et al. [5] in 1975. The fibula, with dual endosteal and periosteal blood circulation, allows for rapid healing of the grafted bone. Although this technique is now widely used clinically, its applications in reconstructing post-traumatic forearm bone defects are limited to a few clinical cases [6].

The MT, also known as the induced-membrane technique, was first reported by Masquelet et al. [7] in 2000. This technique consists of two phases. The first phase (T1) involves thorough bone and soft-tissue debridement and bone cement implementation in the bone defect. The bone cement stimulates the surrounding tissue to form an induced membrane by an allogeneic response. The second phase (T2) usually takes place 4–8 weeks after the bone cement placement in the first phase and involves the removal of the bone cement and bone grafting within the induced membrane. The MT is reportedly an effective therapeutic approach to treating long-bone segmental defects.

Considering the clinical application of FVFG and the MT in treating open forearm fractures accompanied by segmental bone defects, it is evident that there is a conspicuous lack of direct comparative studies examining their radiological and clinical outcomes. Our retrospective cohort study endeavors to fill this research gap by comparing the efficacies of both treatment modalities. The ultimate objective is to give clinicians more precise recommendations based on our research findings. As emphasized in previous literature [8], no consensus has been reached regarding the optimal management of these fractures. Therefore, our investigation thoroughly examines cases treated at a tertiary trauma center spanning the past 15 years. Through this endeavor, we aim to assess and contrast the outcomes associated with FVFG and the MT in reconstructing segmental bone defects resulting from open forearm fractures.

## Materials and methods

### Demographic information gathering—patient characteristics

The current retrospective cohort study included patients admitted to a local hospital between 2005 and 2021 with open forearm fractures with a bone defect longer than 2 cm who underwent FVFG or the MT for bone defect reconstruction. The study excluded patients under 18, those with malignancy or other non-traumatic causes of a segmental bone defect, those with traumatic bone defects less than 2 cm long, and those with multiple organ injuries.

We conducted a retrospective analysis of patients presenting with consecutive open forearm fractures accompanied by a bone defect who underwent reconstruction using either FVFG or the MT at a referral center between 2005 and 2021.

The inclusion criteria were adults (≥ 18 years) with open forearm fractures with a bone defect longer than or equal to 2 cm treated with FVFG or the MT and with a minimum follow-up of 1 year.

The exclusion criteria were patients under 18; individuals diagnosed with malignancy or presenting with segmental bone defects resulting from non-traumatic etiologies; patients with traumatic bone defects shorter than 2 cm in length; those who underwent conventional bone grafting, distraction osteogenesis, or alternative techniques; and patients suffering from multiple organ injuries.

A total of 43 patients were screened out and divided into an FVFG group (18 cases) and an MT group (25 cases) according to the treatment for the bone defect. All patients received appropriate initial treatment in an emergency setting, and the bone defect was reconstructed after general stabilization. The general information is listed in Table 1. There were 24 male and 19 female cases with a mean age of 40.07 years (20–61). Regarding the causes of injury, 22 cases were injured by machine strangulation. Ten cases were hurt by heavy weight smashing. Eleven cases were hit by cars in road accidents. According to the Gustilo–Anderson classification, there were nine cases of grade IIIA, 27 cases of grade IIIB, and seven cases of grade IIIC. In 14 cases, only the radius was involved. In 21 cases, the ulna was involved exclusively. In eight cases, both bones were involved. Bone defects were classified using the long-bone defect typology system proposed by Tetsworth et al. [9]. Ten cases were classified as D3 (A) and 33 as D3 (B). The length of the bone defect ranged from 2.6 to 7.4 cm, with a mean of 4.84 cm. Before 2013, the local institute had treated most cases using the FVFG technique. After 2013, as the Masquelet technique had matured, segmental bone defects were primarily treated using the MT.

**Table 1:** Patient demographics

	Overall	FVFG	MT	*p* value
*N*	43	18	25	
Age (y) (mean ± SD)	42.07 ± 8.95	39.22 ± 7.98	44.12 ± 9.20	0.076
Sex				0.564
Male	24	11 (61.1%)	13 (52.0%)	
Female	19	7 (38.9%)	12 (48%)	
BMI (kg/m^2^) (mean ± SD)	24.86 ± 2.18	25.16 ± 1.70	24.64 ± 2.48	0.444
Mechanism of injury				0.565
Machine injury	22	9 (50%)	13 (52%)	
Smashed by heavy weight	10	3 (16.7%)	7 (28.0%)	
Traffic accident	11	6 (33.3%)	5 (20%)	
Gustilo type				0.936
IIIA	9	3 (16.7%)	6 (24%)	
IIIB	27	13 (72.2%)	14 (56%)	
IIIC	7	2 (11.1%)	5 (20.0%)	
Bone defect site				0.835
Radius	14	6 (33.3%)	8 (32.0%)	
Ulna	21	9 (50%)	12 (48%)	
Radius + ulna	8	3 (16.7%)	5 (20%)	
Defect size (cm) (mean ± SD)	4.84 ± 1.26	5.23 ± 1.18	4.56 ± 1.27	0.085
Defect type				0.115
D3(A)	10	2 (11.1%)	8 (32.0%)	
D3(B)	33	16 (88.9%)	17 (68%)	
D3(C)	0	0	0	
Initial fixation				0.019*
External fixation	22	13 (72.2%)	9 (36%)	
Internal fixation	21	5 (27.8%)	16 (64%)	
Wound coverage				0.852
Direct closure	7	3 (16.7%)	4 (16%)	
Skin graft	17	7 (38.9%)	10 (40%)	
Local flap	4	1 (5.6%)	3 (12%)	
Free flap	15	7 (38.9%)	8 (32%)	
Duration of wound coverage (days) (mean ± SD)	7.49 ± 3.02	9.28 ± 3.29	6.20 ± 2.04	0.000*
Bone defect reconstruction operation time (min) (mean ± SD)	190.70 ± 97.75	284.44 ± 82.55	123.20 ± 23.58	0.000*
Hospital length of stay for bone defect reconstruction (days) (mean ± SD)	10.72 ± 2.38	12.67 ± 1.81	9.32 ± 1.65	0.000*
Bone defect reconstruction hemorrhage (ml)	154.65 ± 73.04	219.44 ± 54.61	108.00 ± 42.52	0.000*
Operation time	3.79 ± 0.77	3.50 ± 0.62	4.00 ± 8.16	0.035*
Comorbidity				
Hypertension	5	2 (11.1%)	3 (12%)	0.931
Diabetes	4	2 (11.1%)	2 (8%)	0.736
Smoking	8	4 (22.2%)	4 (16.0%)	0.615

*FVFG* free vascularized fibular grafts, *MT* Masquelet technique

The study was approved by the Ethics Committee of the local institute (no. LW20220053), and all patients signed the informed consent form. This study was registered on https://www.chictr.org.cn in accordance with the World Medical Association’s Declaration of Helsinki 2013. It is also in accordance with the STROBE reporting checklist [10].

### Initial treatment

After admission, vital sign monitoring and anti-shock treatment were routinely employed. Tetanus immunoglobulin and antibiotics (cefazolin sodium pentahydrate 1.0 g or cefuroxime 1.5 g every 8 h) were injected as a precaution. Brachial plexus nerve block anesthesia or general anesthesia was administered to the patients. A thorough debridement was conducted layer by layer from the skin to the fractured ends. Contaminated tissues with poor blood supply were dissected. After debridement, internal fixation with a plate was carried out for wounds with adequate soft-tissue coverage. When there was insufficient soft-tissue coverage at the fracture site, external fixation was used, followed by vacuum sealing drainage (VSD, Wuhan VSD Medical Science & Technology Co., Ltd., Wuhan, China). Postoperative prophylactic antibiotics were routinely administered for 72 h.

### Bone defect reconstruction

*FVFG group*. One-stage reconstruction was performed when the wound was clean or when a single composite bony flap could cover the wound. However, a two-stage reconstruction was performed in the case of extensive injury and wound infection; i.e., soft-tissue reconstruction was performed first, and then FVFG was performed after the wound had stabilized.

Postoperatively, patients were administered prophylactic low-molecular-weight heparin as part of the routine anticoagulation therapy. They were advised to undergo strict bed rest for 1 week. For patients presenting with skin defects necessitating flap coverage, we also procured a fibular flap besides the fibular graft. Notably, the cutaneous component of the composite graft functioned as an observation window to assess vascular patency.

*MT group*. In the first step, radical debridement of all injured tissues and the implementation of PMMA bone cement spacers (Palacos^®^, Heraeus Kulzer GmbH) in the segmental bone defect were conducted. Masquelet stage II surgery was performed 6–8 weeks later when the wound was completely healed, and the white blood cell count, erythrocyte sedimentation rate, and C-reactive protein were all within normal limits. The induction membrane around the cement was carefully incised and protected to preserve its biological activity. The cement was removed and replaced with autologous iliac bone. The induced membrane was then sutured. Definitive internal fixation was performed on an emergency basis or at the time of wound coverage.

### Follow-up and efficacy evaluation

All patients were followed up regularly at 1, 2, 3, 6, and 12 months after surgery and annually after that. Bone healing, limb function, and complications were monitored. Clinical bone healing was judged according to the method of Commeil et al. [11] based on radiographs. Bone was considered healed if three bone bridges were visible at two CT slides, and the time of bone healing was recorded. Non-healing was defined as bone non-union or limited healing progress observed on X-ray or CT when the patient stopped their treatment at our institution after more than 1 year of follow-up. The diagnosis of postoperative infection was based on the definition of fracture-associated infection [12], clinical symptoms, and laboratory tests. Clinical signs of postoperative infection include localized redness and swelling of the surgical wound, pus or pus-like drainage, unexplained postoperative fever, poor wound healing, and sinus tract formation in the surgical wound, requiring debridement and intravenous antibiotics. Laboratory tests included leukocytosis, elevated C-reactive protein levels, and elevated sedimentation erythrocyte sedimentation rate (ESR) [13].

**Fig. 1:**
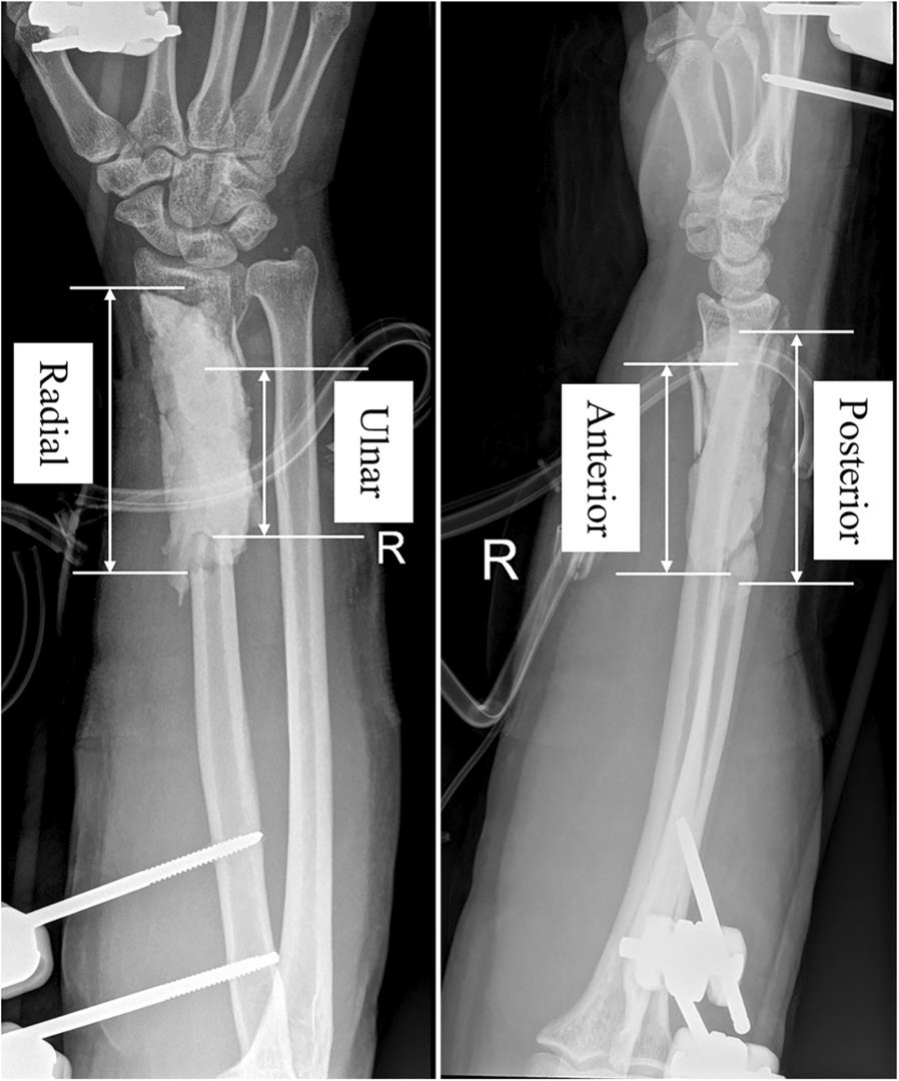
The size of the cortical gap was measured at the perimeter of the cortical bone on the radial, ulnar, anterior, and posterior cortical projections on anteroposterior and lateral radiographs, based on the method reported by Haines et al. [14]. These measurements were averaged over four cortices to obtain the radiographic apparent bone gap for each fracture

The bone defect size was defined as the average length of the largest cortical defect observed in the anterior–posterior and lateral radiographic views, based on the method reported by Haines et al. [14], as shown in Fig. 1. Functional outcome was assessed postoperatively according to the QuickDASH score [15].

### Statistical methods

Statistical analyses were performed using SPSS 26.0 statistical software (SPSS Inc., USA). Correlations between categorical variables were analyzed using the chi-square test or Fisher's exact test. The independent* t*-test or Wilcoxon rank-sum test was used to compare means of continuous variables, and the Mann–Whitney* U* test was used to compare medians of continuous variables. The Shapiro–Wilk test was used to test whether the data were normally distributed. *p* < 0.05 was considered to indicate a statistically significant difference.

## Results

Forty-three patients were followed up for 18–190 months, with a mean of 46.93 months. The mean follow-up time was significantly longer in the FVFG group than in the MT group (74.78 ± 41.11 months versus 26.88 ± 6.03 months, *p* = 0.000). Bone healing time was 3–16 months, with a mean of 4.67 months. The QuickDASH score at the last follow-up was 0–38.6, with a mean value of 17.71. No significant difference between the two groups was observed.

Twenty-two cases were fixed by external fixation in the first stage of treatment, while for the other 21 cases, internal fixation was employed directly. As for the wound coverage, seven cases were directly closed. Skin grafts were used in 17 cases. Local and free flaps were adopted in four and 15 cases, respectively. The average duration of wound coverage was 7.49 days, ranging from 3 to 17 days. The average operating time for bone defect reconstruction was 190.70 ± 97.75 min, the average hospital stay for bone defect reconstruction was 10.72 ± 2.38 days, the average intraoperative bleeding for bone defect reconstruction was 154.65 ± 73.04 ml, and the average number of operations was 3.79 ± 0.77. Operative time, hospital stay, and intraoperative bleeding for bone defect reconstruction were higher in the FVFG group compared to the MT group (284.44 ± 82.55 min versus 123.20 ± 23.58 min, *p* = 0.000; 12.67 ± 1.81 days versus 9.32 ± 1.65 days, *p* = 0.000; 219.44 ± 54.61 ml versus 108.00 ± 42.52 ml, *p* = 0.000), while the number of procedures was lower in both the FVFG group than in the MT group (3.50 ± 0.62 versus 4.00 ± 8.16, *p* = 0.035).

**Fig. 2:**
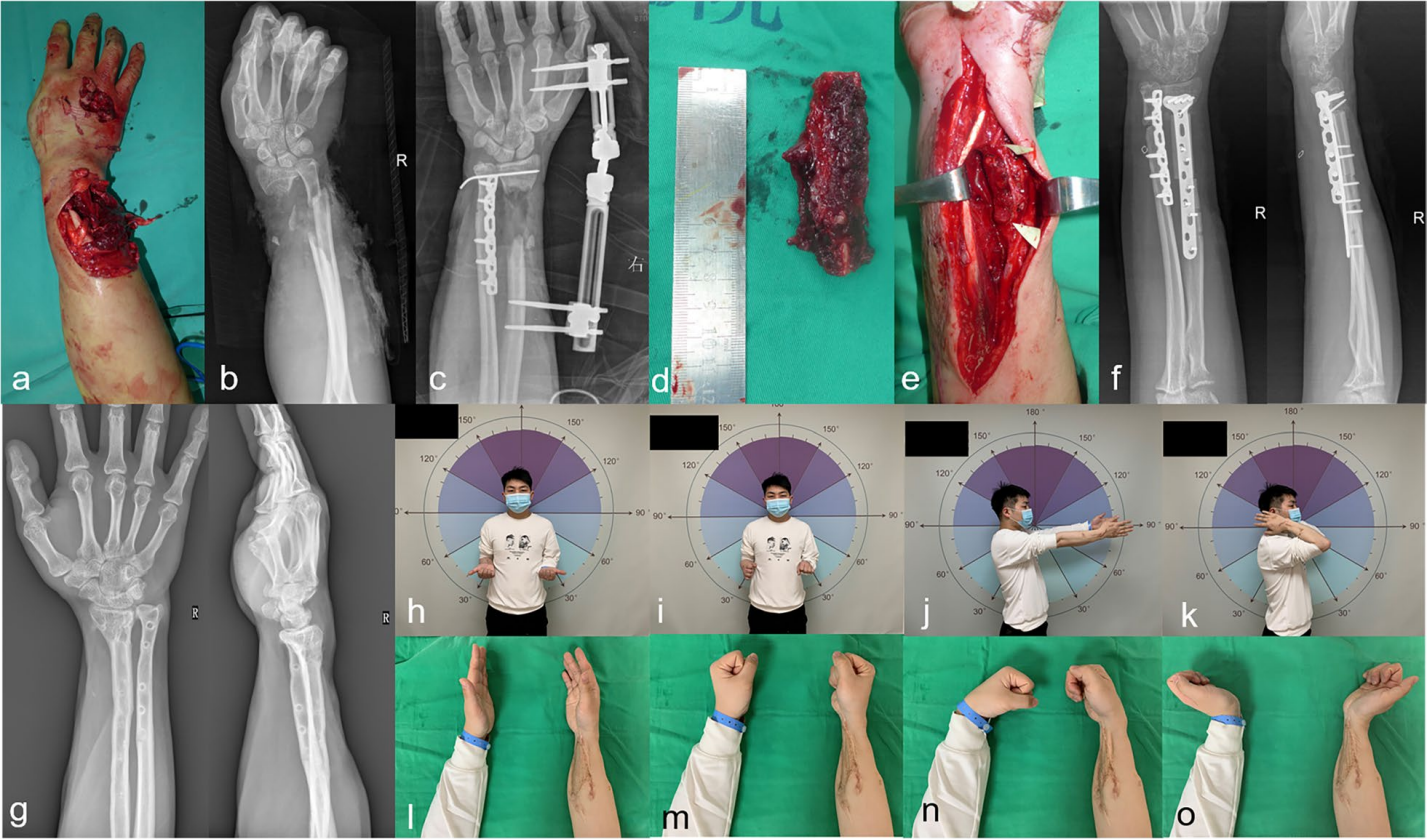
Case 1. **a**–**c** Male, 26 years old, with an open distal forearm fracture (Gustilo IIIA) caused by machine strangulation. He was treated with emergency debridement, internal fixation of the ulna, external fixation of the radius, and tendon repair. **d**–**f** The radial bone defect was treated with a free vascularized fibular graft 2 months after primary surgery. **g** Four years after surgery, with good bone healing. **h**–**o** Last follow-up, with satisfactory function. QuickDASH: 2.3

**Fig. 3:**
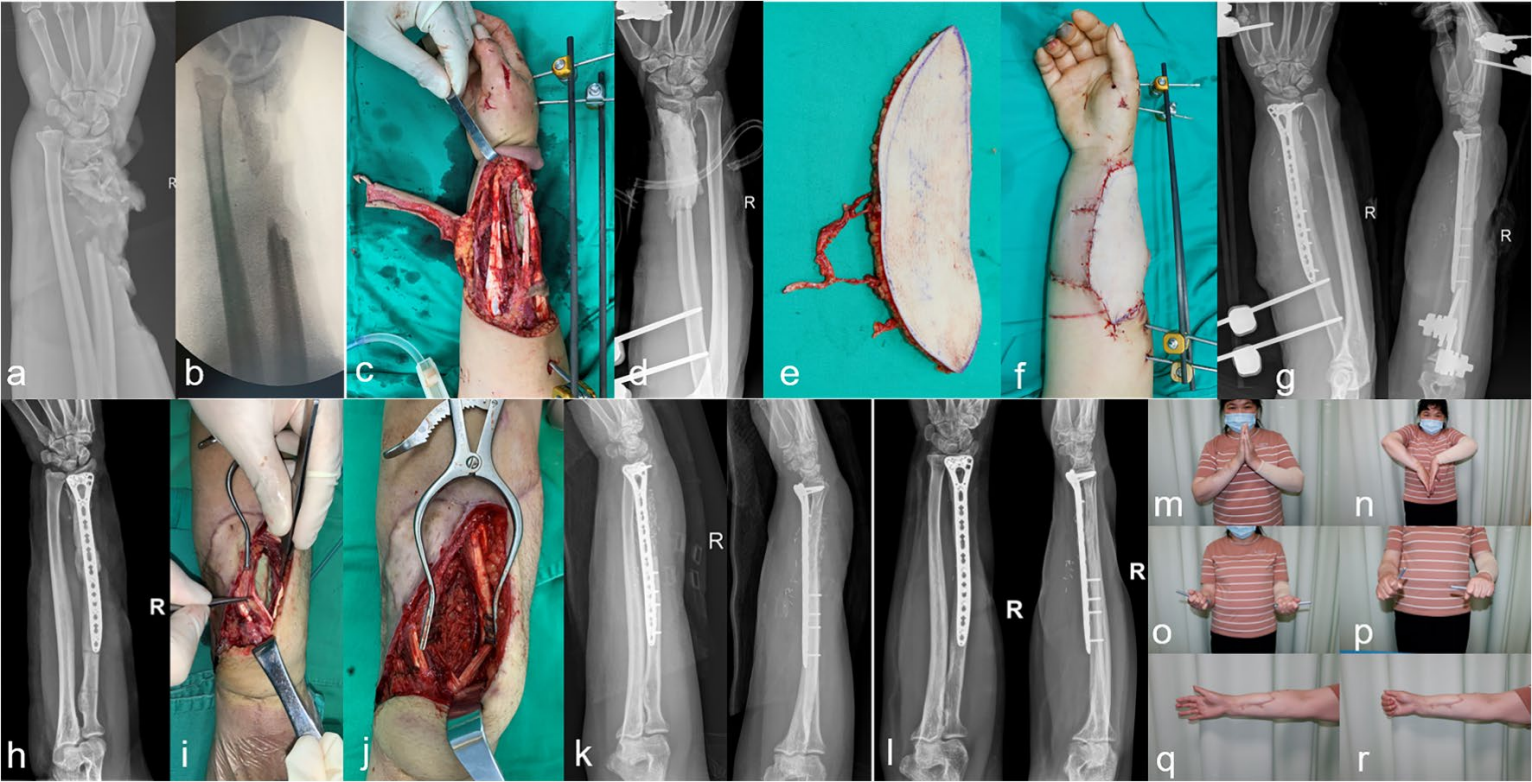
Case 2. **a**–**d** Female, 50 years old, with an open fracture of the right distal radius (Gustilo IIIB) caused by machine strangulation. She was treated with emergency debridement, external fixation, and tendon and vascular repair. The bone defect was filled with bone cement. **e**–**g** Seventy-two hours later, a free anterolateral femoral flap was performed to cover the wound while the radius was internally fixed. **h**–**k** Two months after surgery, the cement was removed and replaced with autologous iliac bone. **i** Two years after stage II of the MT, with bone healing. **m**–**r** Last follow-up, with satisfactory function. QuickDASH: 9.1

Complications were observed in six cases, including one bone non-union in the FVFG group. The MT group contained two cases of deep infection, one case of bone non-union, and two instances of bone bridge formation between the ulnar and radial bones. In cases of deep infection, we conducted a comprehensive debridement and administered sensitive antibiotics for treatment. In the event of non-union, we opted for an additional autologous cancellous bone graft to address the bone non-union. The radioulnar synostosis was treated conservatively. No donor-site complications were observed in the FVFG group, while there were two donor-site complications in the MT group and one case of lateral femoral cutaneous nerve injury. The numbness disappeared 6 months after surgery, and one case of hematoma improved with conservative treatment. The follow-up results are shown in Table 2. Typical cases are shown in Figs. 2–3.

**Table 2:** Follow-up and clinical outcomes

	Overall	FVFG	MT	*p* value
Follow-up (months)	46.93 ± 35.78	74.78 ± 41.11	26.88 ± 6.03	0.000*
Union time (months)	4.67 ± 2.53	4.39 ± 2.48	4.88 ± 2.60	0.841
Quick DASH	17.71 ± 9.68	17.05 ± 10.43	18.18 ± 9.29	0.710
Recipient-site complications	6	1(5.6%)	5(20%)	0.186
Deep infection	2	0	2	
Nonunion	2	1	1	
Radioulnar synostosis	2	0	2	
Donor-site complications	2	0	2 (8%)	0.229
Temporary sensory disturbance		0	1	
Hematomas		0	1	

*FVFG* free vascularized fibular grafts, *MT* Masquelet technique

## Discussion

In this study, we evaluated and compared the radiological and clinical outcomes of segmental bone defects after the reconstruction of open forearm fractures using FVFG and the MT over the past 15 years and found that both FVFG and the MT showed satisfactory clinical outcomes, with a shorter operative time, a shorter length of stay, less intraoperative bleeding, and a slightly higher number of procedures required with the Masquelet technique compared with FVFG.

Open forearm fractures due to high-energy injuries often result in comminuted or multisegmented fractures. Free bone masses are frequently removed during the first debridement stage, resulting in forearm bone defects that are clinically challenging for surgeons [16]. Based on animal experiments, Schmitz et al. [17] defined bone defects with a length greater than 1.5 times the diameter of the long bone as critical bone defects that required surgical treatment.

Currently, the main therapeutic strategies for bone defects include free bone grafting, bone flap grafting with blood vessels, bone transport techniques, and the MT [18, 19]. Due to bone resorption after free bone grafting [20], the traditional indications for free bone grafting are limited to bone defects less than 2 cm in length [21, 22]. Bone transport techniques are typically associated with complex external fixation systems that are susceptible to pin tract infections, pain, prolonged treatment durations, and non-union. Consequently, their application in the upper extremity remains restricted [23, 24]. Revision surgery rates of up to 23.8% have been reported in the literature [25]. In addition, external fixation can harm the mental health of patients, and this harm may persist even after the removal of the external fixator [26]. Therefore, new techniques for treating bone defects are constantly being explored.

Vascularized bone grafts are widely used in bone reconstruction surgery. They can be obtained from the fibula, ilium, ribs, radius, ulna, scapula, femur, humerus, pubic bone, or metatarsus [27, 28]. Vascularized bone grafts help provide nutrients to the deep structures of the graft and achieve stable bone healing, thus allowing for early limb movement and functional recovery. The most widely used technique is FVFG, which can be used in many sites [29]. FVFG has been an irreplaceable method for the reconstruction of large segmental bone defects since the classic FVFG technique was reported by Taylor et al. [5] in 1975, and cases of defects up to 22 cm in length have been reported in the literature [30]. Fixed vascular tips have vessels of a sufficient caliber (2–4 mm in diameter) to allow tension-free anastomosis for the blood supply to the endosteum and periosteum. One of the main benefits of FVFG for the forearm is that it enables one or both forearm bones to be replaced in a single procedure; it also allows the coverage of soft-tissue defects in patients who have experienced complex trauma or infected areas [27]. In addition, the fibula is suitable for bone reconstruction in this anatomical region because it resembles the forearm bone in shape and diameter [31]. Noaman [32] reported 16 upper limb bone defects that were treated with FVFG and had a mean healing time of 3.5 months. Cano-Luís et al. [6] reported long-term results of 14 forearm bone defects that were treated with FVFG and had a mean healing time of 4.2 months. FVFG has been used in our institution since the early 1990s. The time to bone healing in the FVFG group of patients in this study was 3.7 months, similar to what has been reported in the literature. One case with diabetes mellitus had bone non-union, which may be attributed to the poor blood supply to the grafted bone site due to occlusion of the FVFG vessels.

In the case of FVFG, donor-site morbidity is one of the significant issues. The common complications of fibular harvest include neurovascular injury, compartment syndrome, extensor dysfunction, abductor weakness, and ankle instability. However, no such complications were observed in this case series, which may be partly related to the small number of cases.

Since its initial publication in 2000, the MT has had relatively modest technical requirements and was initially utilized to treat infected bone defects in long bones. [33, 34]. Basic studies [35, 36] have shown that the induced membranes produced after cement filling are rich in several cytokines, including vascular endothelial growth factor and bone morphogenetic protein-2 (BMP-2), which can promote new bone formation. In the literature, the application of the MT for post-traumatic bone defects has been reported, with bone healing rates of more than 90% (up to 100% in some cases) [20, 37] and relatively low rates of bone resorption [38]. Although the MT has been proposed for over 2 decades [7], it was first used in our institution in 2013 [39]. Current studies on MT techniques have focused on the lower extremity, and there are few studies on the application of the MT to the upper extremity, especially forearm bone defects [18, 40]. Luo et al. [41] reported seven patients with infected bone defects in the forearm—the mean radial defect length was 5.8 cm and the mean ulnar defect length was 5.5 cm—which were treated with the MT, and all patients showed bone healing.

Walker et al. [42] applied the MT to successfully treat nine cases of post-traumatic bone defects in the forearm with a mean bone defect length of 4.7 cm, including five acute open fractures and four cases of bone non-union. In this study, we treated 25 cases of segmental bone defects of the forearm using the MT, and all patients healed well except for two cases of infection and one case of bone non-union. We believe that filling the bone defect with bone cement at the emergency stage for open forearm fractures can fill the dead space, prevent fibrous tissue from growing, and stimulate the surrounding tissue to induce membrane formation. Besides, bone cement can maintain the stability of the fracture end and avoid internal fixation failure [43, 44]. In this study, two other patients in the MT group who had bone defects in both the ulnar and radial bones had postoperative bone bridge formation between the ulnar and radial bones, which may be related to the destruction of the interosseous membrane between these bones and the excessive volume of bone cement filling.

Although FVFG and the MT have been widely used clinically with good prognoses (Table 3), a comparison between the two techniques when they are applied in the forearm has not been performed [52]. Wen et al. [53] compared three treatment modalities (free vascularized fibular graft, distraction osteogenesis, and the Masquelet technique) for extended bone defects after lower-extremity trauma and showed similar clinical outcomes for all three modalities. Lan et al. [54] compared the results of FVFG and the MT for the reconstruction of Gustilo III tibial fractures. They found that FVFG has greater potential for reconstructing larger bone defects. In contrast, the MT may be essential in smaller bone defects, severe surgical site infections, and osteomyelitis. A recent review [55] has shown that one of the most recent developments in massive allograft reconstruction post-tumor resection does not attempt to mimic a vascularized bone flap. Instead, the focus is on osteointegration of the allograft bone segment ends. The bulk of the graft is shielded with PMMA to prevent graft resorption. They believe that the Masquelet technique is safe, cheap, and, most importantly, effective. Singh et al. [52] conducted a meta-analysis by separately searching for studies on treating upper-extremity bone defects using vascularized bone grafting (VBG) and the Masquelet technique. Their findings indicate no statistically significant difference in union rates between VBG and the Masquelet technique for upper-extremity bone defects, regardless of the defect size. These studies highlight the shift in our surgical approach from the previous predominance of FVFG to the current predominance of the MT technique. We found that the MT technique had a shorter operative time, was less technically challenging, and did not significantly increase bone healing time. The results confirm our clinical observations, and with the increased experience accrued during the study period, we have established a standardized team and approach to these cases [2, 56], reducing complications and operative times. Therefore, segmental bone defects have mainly been treated with the MT since it started to be used in our institution in 2013.

**Table 3:** Literature review of the outcome studies regarding FVFG and the MT

Author	Year	Cases,* n*	Mean defect size, cm (range)	Mean time to union, months (range)	Reported union,* n* (%)	Mean follow up, months (range)	Complications,* n* (%)
FVFG							
Dell [45]	1984	4	N/A	N/A	3 (75%)	N/A	0
Olekas [46]	1991	15	9.3 (5–12)	N/A	11 (73.3%)	23.5 (7–60)	4 (26.7%)
Jupiter [47]	1997	9	7.9 (4.5–11)	N/A	8 (88.9%)	24	3 (33.3%)
Adani [31]	2004	12	8.5 (6–13)	4.8 (2.5–8)	11 (91.6%)	N/A (10–93)	3 (25%)
Safoury [48]	2005	18	N/A	4 (N/A)	17 (94.4%)	N/A	N/A
Cano-Luís [6]	2018	14	N/A (6–11)	4.3 (2.5–7)	13 (92.9%)	166.8 (96–276)	4 (28.58%)
MT							
Luo [41]	2017	7	5.6 (4–8)	N/A	7 (100%)	86.7 (41–150)	4 (57.14%)
Dhar [49]	2019	12	5 (3.5–7)	7.8 (6–12)	12 (100%)	N/A	0
Walker [42]	2019	9	3.9 (1.7–5.4)	4.7 (3–12)	9 (100%)	4.7 (3–12)	1 (11.11%)
Bourgeois [50]	2020	6	6.4 (4.8–11.0)	4 (2.3–6.3)	5 (83.3%)	103.4 (67.4–144.3)	3 (50%)
Commeil [11]	2021	10	4.3 (2–8)	9.2 (4–13)	9 (90%)	50.3 (14–74)	1 (10%)
Lauthe [51]	2021	13	4 (0–12)	5 (3–8)	12 (92.3%)	30	2 (15.38%)

*FVFG* free vascularized fibular grafts, *MT* Masquelet technique

This study has several limitations. Firstly, its retrospective and non-randomized design constrained the data collection scope. Secondly, the small sample size hinders the generalizability of the findings. Additionally, each bone defect reconstruction was unique, with the defect size determining the reconstruction scale, making it challenging to control all relevant variables and identify comparable scenarios. Furthermore, the reconstructions were performed at a single institution, potentially limiting the applicability of the results. Finally, the varying follow-up periods may obscure crucial long-term differences. Therefore, prospective studies with larger samples are essential to validate the study's conclusions.

## Conclusions

FVFG and the MT have shown satisfactory clinical results when used to treat forearm segmental bone defects, with a decreased operative time, hospital stay, and intraoperative bleeding seen in the MT group compared to the FVFG group. Although the number of procedures was higher in the MT group than in the FVFG group, the technical difficulty of the MT was lower. Considering the complexity and unpredictability of trauma, a flexible treatment strategy or even a combination of both approaches for the reconstruction of segmental forearm defects may achieve satisfactory outcomes.

## Supplementary Information

Supplementary Material 1. Appendix 1. Preoperative 3D CT scans of both the donor and recipient sites to accurately determine the necessary bone graft volume in case 2: a 3D CT reconstruction of the forearm.b 3D CT reconstruction of the forearm after cement removal. c, d The extent of the anterior iliac bone graft harvest

Supplementary Material 2. Appendix 2. A male patient, 39 years old, with an open fracture of the left ulna (Gustilo IIIA) caused by machine strangulation. a, b Preoperative anteroposterior and lateral radiographs.c, d Post-emergency-surgery anteroposterior and lateral radiographs.e, f Ulna bone defect treated with a free vascularized fibular graft 1 month after primary surgery.g, h Twenty-six months after surgery, with good bone healing

Supplementary Material 3.

## Data Availability

The data that support the findings of this study are available from the corresponding author upon reasonable request.
