# Supplementary figures and images for: Comparison of free vascularized fibular grafts and the Masquelet technique for the treatment of segmental bone defects with open forearm fractures: a retrospective cohort study
Source: J Orthop Traumatol. 2024 Sep 28;25:44. doi: 10.1186/s10195-024-00787-x (PMC11438757; doi:10.1186/s10195-024-00787-x)

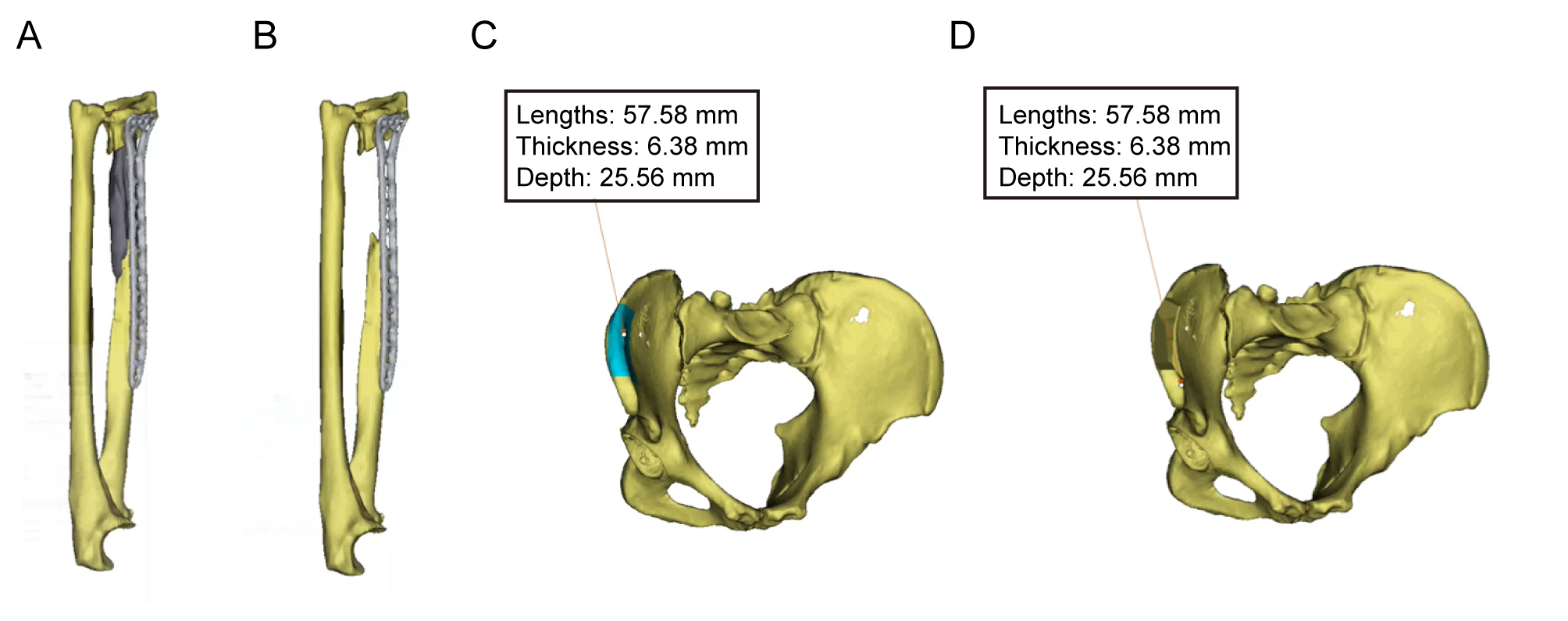

Supplement: Supplementary file 1 — Supplementary Material 1. Appendix 1. Preoperative 3D CT scans of both the donor and recipient sites to accurately determine the necessary bone graft volume in case 2: a 3D CT reconstruction of the forearm.b 3D CT reconstruction of the forearm after cement removal. c, d The extent of the anterior iliac bone graft harvest [file 10195_2024_787_MOESM1_ESM.tif]

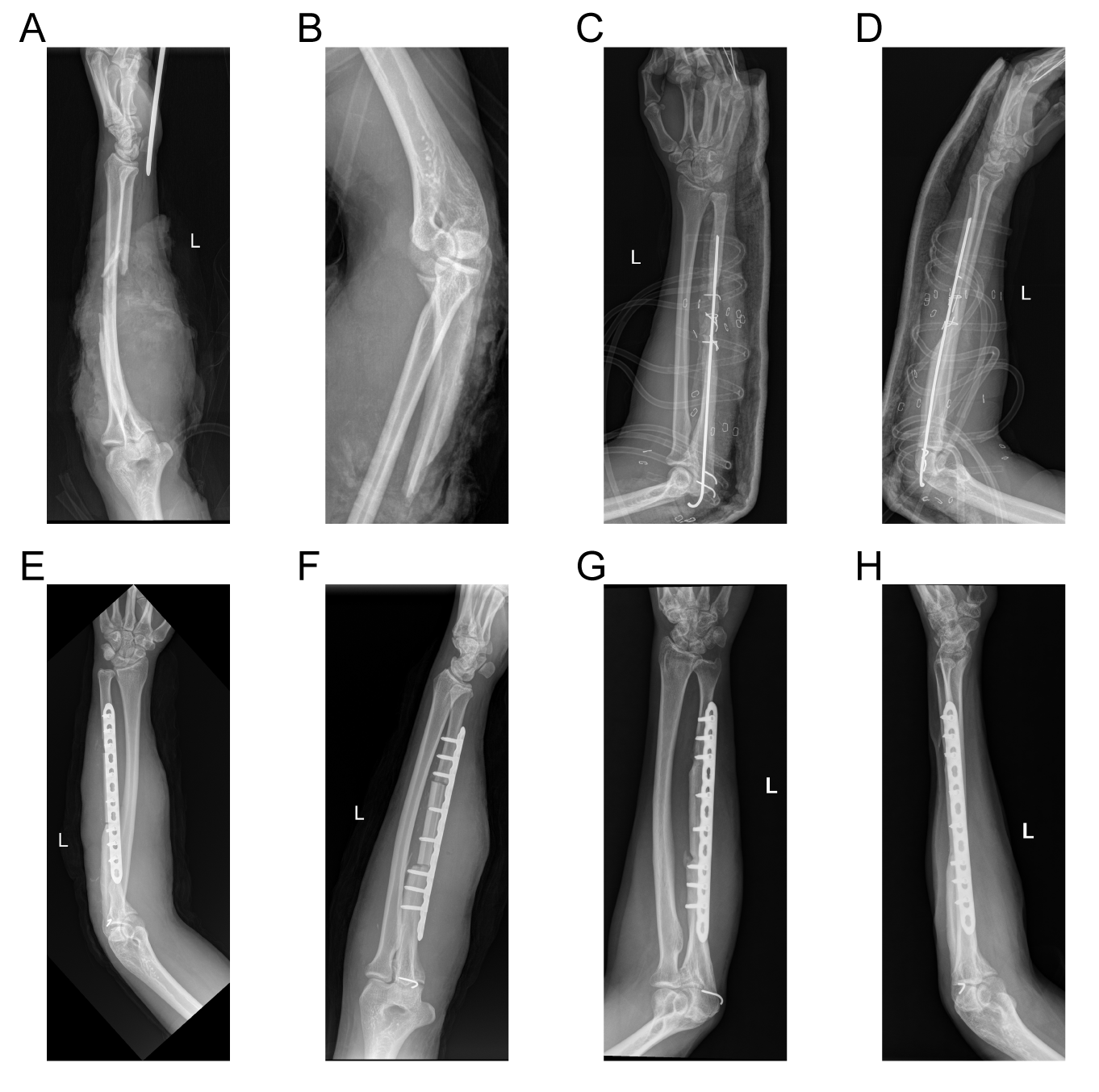

Supplement: Supplementary file 2 — Supplementary Material 2. Appendix 2. A male patient, 39 years old, with an open fracture of the left ulna (Gustilo IIIA) caused by machine strangulation. a, b Preoperative anteroposterior and lateral radiographs.c, d Post-emergency-surgery anteroposterior and lateral radiographs.e, f Ulna bone defect treated with a free vascularized fibular graft 1 month after primary surgery.g, h Twenty-six months after surgery, with good bone healing [file 10195_2024_787_MOESM2_ESM.tif]
